# The effect of social pension on health-related quality of life of the rural older people: a panel study from China

**DOI:** 10.1186/s12877-024-04880-y

**Published:** 2024-03-27

**Authors:** Siyuan Liu, Hong He, Hanzhi Gao

**Affiliations:** 1 Population Development Studies Center, School of Sociology and Population Studies, Renmin University of China, 59 Zhongguancun Street, Haidian District, Beijing, China; 2 Interdisciplinary platform for public health and disease prevention and control of Renmin, Institute of Health Sciences Research, University of China, Renmin University of China, 59 Zhongguancun Street, Haidian District, Beijing, China; 3 School of International Studies, Renmin University of China, 59 Zhongguancun Street, Haidian District, Beijing, China

**Keywords:** Older people, Quality of life, Social welfare programs, Geriatrics, Population health, Well-being, Rural, Inequality, Policy evaluation, Quantitative methods

## Abstract

**Background:**

Social pensions, social assistance systems for older people in rural areas, have been put into place in many nations and have positively impacted health. The long-term health consequences of social pension programs in China are uncertain. The aim of this study is to evaluate the long-term health consequences of the new rural social pension (NRSP) for the rural older people in China.

**Methods:**

Based on the 2011 and 2018 China Health and Retirement Longitudinal Study, we compared the scores on eight Health-Related Quality of Life (HRQoL) subscales of the rural older people before and after participation in the NRSP. The propensity score matching and difference-in-difference methods were used in data analysis. We also conducted a heterogeneity analysis for subgroups with different characteristics and pension enrolment times.

**Results:**

The NRSP significantly enhanced scores on physical functioning, role-physical, and self-rated mental health of old rural participants by 1.90 (*p* < 0.01), 2.05 (*p* < 0.01), and 2.93 (*p* < 0.05), respectively. After excluding newly enrolled individuals, the beneficial health effects of the NRSP remained significant. There were no significant changes due to NRSP in the other five scores on the HRQoL subscale of the rural older people. The NRSP had more health benefits for older people in underdeveloped areas without formal schooling.

**Conclusions:**

The NRSP reduced health disparities and had long-term benefits on the physical and mental health of the rural older people. We suggest continuously expanding the NRSP throughout rural China and further improving the social support system to enhance the overall quality of life of the rural older people. Comparable social pension programs aimed at underprivileged groups could also be conducted in other low- or middle-income nations.

**Supplementary Information:**

The online version contains supplementary material available at 10.1186/s12877-024-04880-y.

## Introduction

The number of the older people is constantly rising as the global population aging process intensifies. By 2030, there will be 1.4 billion people aged 60 years or older in the world, up from 1 billion in 2020 [1]. As the largest developing country, China has one of the world’s fastest-rising older populations. Approximately 264 million people in China were 60 years of age or older by the end of 2020, comprising 18.7% of the country’s entire population [2]. Because human bodily functions tend to deteriorate with advancing age, the older people have a higher risk of developing diseases than young people [3]. The health status of aged individuals and health promotion initiatives aimed at improving the older people’s quality of life have been the focus of many scientists and policymakers worldwide, including in China [4, 5].

Nearly two out of every three older people in China reside in rural areas [6]. The health status of China’s rural older people has received increasing attention in recent years because they experience more severe health issues than urban older people. According to previous studies, older people in rural areas had higher probabilities of disability, poorer self-rated health, and insufficient healthcare utilization than the older people in urban areas [7, 8]. The rural older people have a disproportionately greater prevalence of poverty, worsening healthcare infrastructure, and lower levels of health literacy, which contribute to these health disparities [7, 9]. Therefore, the older people in China’s rural areas have a crucial stake in establishing an efficient social security system [3].

On September 1, 2009, the China State Council issued the “Instruction of Implementing New Rural Social Pension Pilot” to improve the welfare of older people in rural areas and promote social equality [10]. The New Rural Social Pension (NRSP) started in 2009 and achieved universal coverage at the end of 2012. Except for students and those who have enrolled in retirement pensions for urban employees, rural inhabitants who attain 16 years of age can voluntarily join the NRSP in their communities. The NRSP is funded by individual contributions, a collective allowance, the government, business and social organizations, and others. The main distinction between the NRSP and the previous rural social pension introduced in 1992 is that the NRSP combines social pooling and individual accounts rather than a sole individual account. The basic pension and premium subsidy benefits provided by the federal and municipal governments attracted the elderly to sign up for the NRSP [11]. In 2012, 71.6% of rural residents had signed up for the NRSP [12]. The government initially provided NRSP participants over 60 years old with a basic pension of at least CNY 660 (approximately US$ 98) per year. In 2014, the minimum basic pension was raised to CNY 840 (approximately US $123) per year [10]. The registrants could also obtain more money from their contribution accounts in addition to the basic pension. The maximum annual pension reached CNY 7,116 (approximately US$1,062) [10]. Even though around half of the elderly receive only a basic pension, the NRSP’s supplemental income is an essential source of income for the older people Chinese living in rural areas [13]. According to estimates, the NRSP could boost discretionary income and alter the behaviour of insured older people, particularly for low-income groups [14]. Several studies have examined the impact of the NRSP, however, the majority of them concentrated on intergenerational assistance, labour supply and retirement, and patterns of older people care [14–18].

Health implications of social pension are noteworthy. Pensions can impact the physical and emotional health of the older people, according to international experience [19–21]. One study showed that pensions might increase self-rated health and activities of daily living (ADLs) among older people in South Africa [21], and Galiani et al. showed that pensions could reduce the level of depression in Mexican older people by 17% [19]. Several studies have estimated that the NRSP affects the health of older rural Chinese people [3, 13, 22–24]. Ma and Oshio reported that participation in the NRSP was positively associated with self-rated health and cognitive function in low-income older people [24]. However, these studies have shown inconsistent results. For instance, Wang et al. revealed that the NRSP had no appreciable influence on the mental health of the older people living in rural areas in China [23], whereas Xu and Liu revealed that the NRSP has significant and detrimental consequences for mental health among older people [3]. The results of these studies also contain bias. Most of them did not use data from after the NRSP’s universal coverage and did not account for the time impact and self-selection bias [3, 13, 22].

Health-related quality of life (HRQoL) is a multidimensional concept that includes domains related to physical, mental, emotional, and social functioning. Compared with the formerly widely-used measures of older people health (such as ADLs and cognitive levels), HRQoL allows for a thorough assessment of the health consequences of the NRSP from the standpoint of quality of life. Although research from China and Vietnam has demonstrated some established links between pension and HRQoL of the older people [25, 26], no published study has estimated the impact of the NRSP on HRQoL in rural older people.

In addition to narrowing the aforementioned research gaps, more robust information addressing the health effects of the NRSP could aid policymakers in further enhancing older people’s quality of life and bolstering social assistance for the older people in rural areas. Therefore, the objectives of this study are to (1) investigate the influence of the NRSP on the HRQoL of old Chinese rural residents, and (2) assess the variation in policy implications of the NRSP for the older people with various enrolment periods and socioeconomic characteristics.

## Method

### Data

Data was obtained from the China Health and Retirement Longitudinal Study (CHARLS) conducted by the National Development Academy of Peking University [27, 28]. The CHARLS baseline survey (2011) covered 28 of 32 provincial administrative regions, 150 districts, and 450 villages and urban communities in mainland China, and involved 17,780 individuals aged 45 years and above. A stratified multi-stage probability-proportional-to-size (PPS) random sampling strategy was adopted in baseline sampling [27]. The CHARLS sample is highly representative of the Chinese national middle-aged and older population. In the follow-up waves of the survey, the samples were continuously refreshed and reached 19,817 individuals in 2018 [29]. The CHARLS contains a wide range of information, including demographic background, family information, family transfer, health status and functioning, health care and insurance, work, retirement and pension, household income, and individual income. CHARLS includes detailed health indicators, such as ADLs, instrumental ADLs, depression, and cognitive capability. Thus, it has been widely used in studies examining the health and socioeconomic status of the older people in China [30, 31].

**Fig. 1:**
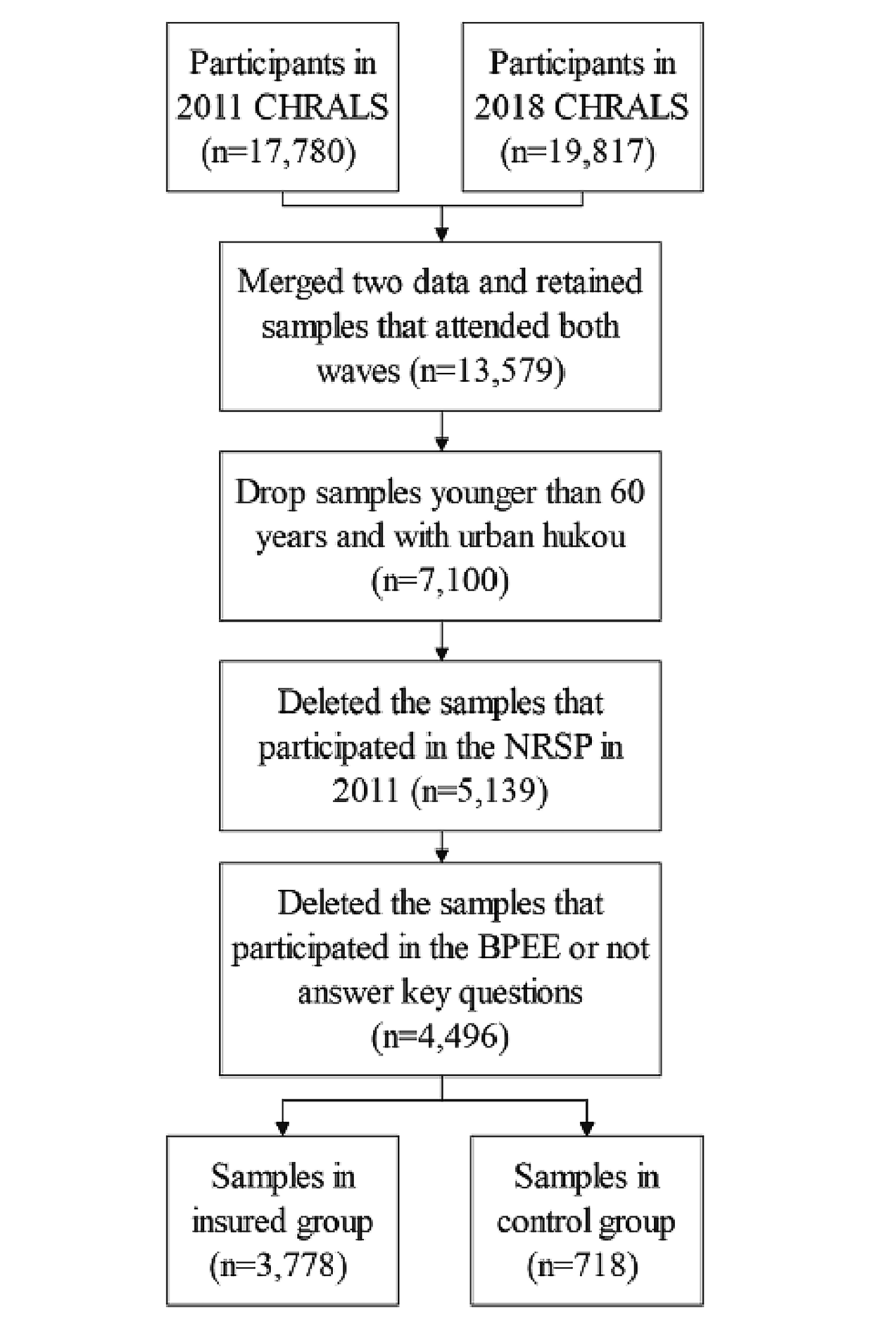
Flow diagram for study participants

To access a considerable number of individuals for evaluating the long-term health implications of the NRSP, we used 2011 and 2018 CHRALS in the analysis. We screened the data and constructed the insured group and the control group. The insured group were individuals who did not participate in the NRSP in 2011 but then enrolled voluntarily before 2018. The control group were individuals who did not participate in the NRSP in 2011. Detailed steps are shown in Fig. 1. We first merged the CHARLS 2011 and 2018 datasets and retained only samples that attended both waves. Based on the research objective of this study, we then drop individuals younger than 60 years and with urban hukou (i.e., a household registration system used in mainland China). To constitute two-phase panel data of the quasi-natural experiment of the NRSP, both the insured and control groups did not participate in the NRSP in 2011. Thus, we deleted the samples that participated in the NRSP in 2011. We also eliminated the individuals who participated in the Basic Pension for Enterprise Employees (BPEE) due to the restriction of enrolment in the NRSP [10]. The individuals who did not answer treatment questions were excluded. After the selection procedure, 4,496 older people were admitted to this study, including 3,778 individuals in the insured group and 718 individuals in the control group.

### Measures

In this study, the outcome variable was the health-related quality of life (HRQoL) of the older people. Based on the most commonly used Short Form 36 (SF-36) and an established new scale [32, 33], we assessed the HRQoL using eight subscales: (1) physical functioning, (2) physical role, (3) bodily pain, (4) general health, (5) vitality, (6) social functioning, (7) emotional role, and (8) mental health. The first four components measure physical health of the older people from perspectives of physical activities, work, daily activities, bodily pain, and personally evaluated health. The last four components measure mental health of the older people from personal feelings, social activities, and emotional interference perspectives. A series of variables from the CHARLS (Additional file STable 1) were used to measure each subscale with scores ranging from 0 to 100 (i.e., a higher score indicates better quality of life). The details of the selection and development of these variables are shown in Hao et al. [32]. They have shown that these scales have good reliability and validity in China.

The treatment variable is a dummy variable indicating “whether older people participated in the new rural social pension or not”. The older people who participated in the NRSP in 2018 were coded as “1”. The older people who did not participate in the NRSP in 2018 were coded as “0”.

Based on previous research [22, 24, 34, 35], we considered a set of control variables that might impact the HRQoL and the decision to enrol in NRSP. The measurements, mean values, and standard deviations of these variables are shown in Table 1. The covariates included some demographic and socioeconomic characteristics of the elderly, such as gender, age, marital status, educational attainment, annual household income, and region. Additionally, we considered the intergenerational relationship and family status (i.e., intergenerational support and number of children alive) as covariates. We also controlled for the medical insurance enrolment (i.e., participation in New Rural Cooperative Medical System) and lifestyle factors (i.e., smoking history, alcohol consumption history, and physical activity).

**Table 1:** Variable measurements and descriptive statistics

Variable	Measurement	Mean	St.Dev.
*Outcome Variables*:			
Physical Functioning	Continuous variable: the score of performing physical activities without limitations due to health	72.02	11.63
Role-Physical	Continuous variable: the score of work or other daily activities without interference due to physical health problems	93.55	14.46
Bodily Pain	Continuous variable: the score of bodily pain	85.81	16.02
General Health	Continuous variable: the score of personal evaluated health	57.57	19.10
Vitality	Continuous variable: the score of feeling pep and energy	78.48	21.95
Social Functioning	Continuous variable: the score of performing normal social activities without interference due to physical or emotional problems	22.83	26.74
Role-Emotional	Continuous variable: the score of work or other daily activities without interference due to emotional problems	72.72	24.69
Mental Health	Continuous variable: the score of felling peaceful, happy, and calm	74.54	18.11
*Treatment Variables*:			
New Rural Social Pension	Dummy variable: 1, receive new rural social pension in 2018; 0, do not receive new rural social pension in 2018	0.84	0.37
*Covariates*:			
Gender	Dummy variable: 1, male; 0, female	0.47	0.50
Age	An individual’s age at survey date	65.46	7.96
Marital Status	Dummy variable: 1, married; 0, otherwise	0.81	0.39
Educational Attainment	Dummy variable: 1, do not have formal education; 0, have formal education	0.37	0.50
Annual Household Income	Total income of all household members at survey year (log)	7.99	2.95
Intergenerational Support	Financial support from children (log)	2.94	3.80
Number of Children Alive	Dummy variable: 1, more than two children alive; 0, less than two children alive	0.65	0.48
Medical Insurance	Dummy variable: 1, participate in new rural cooperative medical system; 0, do not participate in new rural cooperative medical system	0.97	0.18
Smoking History	Dummy variable: 1, have smoke history; 0, do not have smoke history	0.28	0.45
Alcohol Consumption History	Dummy variable: 1, drink alcohol; 0, do not drink alcohol	0.32	0.47
Physical Activity	Dummy variable: 1, do physical activities at least 10 min every day; 0, do not do physical activities at least 10 min every day	0.68	0.46
Region	Dummy variable: 1, eastern area; 0, middle and west area	0.25	0.43

### Analysis

Enrolment in the NRSP is a voluntary choice for the elderly, which can lead to a self-selection problem in assessing the effects of the NRSP on their health status [36]. This self-selection problem may come from observable characteristics such as income, age and marital status; it may also come from unobservable characteristics such as risk appetite. Therefore, to reduce this bias, we adopt the propensity score matching (PSM) and difference-in-differences (DID) methods to evaluate the health impacts of the NRSP [37].

Using the DID method, we can eliminate unobservable individual heterogeneity that does not change with time and eliminate the common trend of insured group and control group in 2011 and 2018. The DID regression model can be written as follows:

1 $$ \begin{gathered}{y_{it}} = {\alpha _0} + \gamma (Treate{d_i} \times Tim{e_i}) + \hfill \\\,\,\,\,\,\,\,\,\,\,\,\,\,{\alpha _1}Treate{d_i} + {\alpha _2}Tim{e_i} + \hfill \\\,\,\,\,\,\,\,\,\,\,\,\,\,\beta {X_{it}} + {\delta _i} + {\lambda _t} + {_{it}} \hfill \\ \end{gathered} $$

In the formula, $$ {y}_{it}$$ is the outcome variable (i.e., the HRQoL). $$ {Treated}_{i}$$ is a dummy variable representing the difference between the insured group and the control group. $$ {Treated}_{i}=1$$ represents individual ($$ i$$) belonging to the insured group, and $$ {Treated}_{i}=0$$ represents individual $$ \left(i\right)$$ belonging to the control group. $$ {Time}_{i}$$ is a dummy variable that represents different times of the surveys. $$ {Time}_{i}=1$$ represents survey year 2018, and $$ {Time}_{i}=0$$ represents survey year 2011. $$ {Treated}_{i}\times {Time}_{i}$$ is the core of the model and represents the treatment effect of the NRSP on the HRQoL. $$ {X}_{it}$$ represents a series of covariates, including gender, age, marital status, educational attainment, annual household income, intergenerational support, number of children alive, medical insurance, smoking history, alcohol consumption history, physical activity, and region. $$ {\delta }_{i}$$ and $${\lambda _t}$$represent the individual and time fixed effect, respectively. $$ {\epsilon }_{it}$$ is the error term. $$ {\alpha }_{0}$$ is a constant parameter. $$ {\alpha }_{1}$$ represents the differences between the insured and control groups in 2011. $$ {\alpha }_{2}$$ represents differences in control group outcomes between 2011 and 2018. $$ \beta$$ represents regression parameters of the control variables. $$ \gamma $$ is the policy effect.

To effectively control for the differences in observable characteristics between the insured group and the control group, we used the PSM-DID method based on the above DID model. Using the PSM method, we selected matched uninsured samples in the control group who were similar to the insured samples in the insured group. The policy effect of the NRSP is the average treatment effect on the treated (ATT) of the insured group. ATT can be expressed as:

2 $$ \begin{gathered}{\text{ATT}} = {\text{E(}}Y_{i,post}^P - Y_{i,pre}^P|{D_i} = 1) - \hfill \\\,\,\,\,\,\,\,\,\,\,\,\,\,\,\,\,\,\,{\text{E}}\left( {Y_{i,post}^{NP} - Y_{i,pre}^{NP}|{D_i} = 1} \right) \hfill \\ \end{gathered}$$

In the formula, $$ {Y}_{i,pre}^{P}$$ and $$ {Y}_{i,post}^{P}$$ represent the potential pre-treatment and post-treatment outcomes, respectively, in the case of individual ($$ i$$) participating in the NRSP (P). $$ {Y}_{i,pre}^{NP}$$ and $$ {Y}_{i,post}^{NP}$$ represent the potential pre-treatment and post-treatment outcomes, respectively, in the case of individual ($$ i$$) not participating in the NRSP (NP). $$ {D}_{i}$$ is a binary dummy variable, $$ {D}_{i}=1$$ represents participation in the NRSP, and $$ {D}_{i}=0$$ represents non-participation in the NRSP.

Due to that ${\text{E(}}Y_{i,post}^{NP} - Y_{i,pre}^{NP}|{D_i} = 1)$is unobservable in estimation, simply using ${\text{E(}}Y_{i,post}^{NP} - Y_{i,pre}^{NP}|{D_i} = 0)$as an alternative would lead to selection bias. Thus, in this study, we estimated ATT using the following formula [38, 39]:

3 $$ \begin{gathered}{\text{ATT}} = {{\text{E}}_{P\left( {{X_i}} \right)|{D_i} = 1}}\{ E(Y_{i,post}^P - Y_{i,pre}^P|P\left( {{X_i}} \right),{D_i} = 1) \hfill \\\,\,\,\,\,\,\,\,\,\,\,\,\,\,\,\,\,\,\,\,\,\,\, - {\text{E}}\left( {Y_{i,post}^{NP} - Y_{i,pre}^{NP}|P\left( {{X_i}} \right),{D_i} = 0} \right)\} \hfill \\ \end{gathered}$$

In the formula, $$ P\left({X}_{i}\right)=\text{P}\text{r}({D}_{i}=1|{X}_{i})$$ is a propensity score function, namely the probability of individual $$ i$$ participating in the NRSP in the case of a given “a set of observable characteristic X”. These characteristics included gender, age, marital status, educational attainment, annual household income, intergenerational support, number of children alive, medical insurance, smoking history, alcohol consumption history, physical activity, and region.

We chose the logit model when estimating the propensity score function. The explained variable is $$ {D}_{i}$$. The explanatory variables are the variables that affect whether individual $$ i$$ participates in the NRSP $$ {D}_{i}$$ and the health status of the elderly$$ {Y}_{i}$$. These variables include socioeconomic characteristics, intergenerational relationships, and medical insurance participation. We adopted the most commonly used Kernel matching method, with Gaussian functions as kernel functions and 0.06 as the bandwidth [16, 32]. After estimating the propensity score of each sample, we matched the samples by choosing those individuals who fell within the “common support” propensity score range and matching one or more control samples who were “close enough” for each insured sample. An analysis of the validity of the common support consumption (i.e., matching successfully retained adequate samples with similar characteristics) was conducted as suggested [40]. We also conducted a balance test to check the changes in the distribution of the relevant covariates in both the insured and control groups following previous studies [39]. To better reflect the sensitivity and reliability of the results, we conducted a robustness check using different matching methods (k-nearest neighbours matching and radius matching) and bandwidths (0.1, 0.08, 0.04, 0.02).

## Results

### Descriptive statistics

Table 2 shows the HRQoL of the rural older people in the insured and control group in 2011 and 2018. The Chinese rural older people had relatively higher role-physical and bodily pain scores, ranging from 81.24 to 95.69. The physical functioning, vitality, role-emotional, and mental health scores ranged from 69.95 to 81.12. The social functioning and general health scores of the elderly were relatively low (ranging from 22.48 to 59.83).

**Table 2:** Descriptive statistics of the health-related quality of life of the rural elderly in China

	2011		2018	
Variables	Insured Group	Control Group	Insured Group	Control Group
Physical Functioning	73.48*** (10.67)	74.62 (10.44)	70.47 (11.98)	69.95 (14.02)
Role-Physical	95.35 (12.35)	95.69 (12.50)	92.00*** (15.41)	90.15 (19.12)
Bodily Pain	89.87** (14.22)	91.08 (13.60)	81.24*** (16.77)	83.33 (15.33)
General Health	57.34*** (17.74)	59.83 (18.70)	57.10** (20.29)	58.87 (20.26)
Vitality	79.57* (20.98)	81.12 (20.26)	76.72** (23.15)	78.88 (21.93)
Social Functioning	22.93 (26.61)	24.14 (26.82)	22.48 (26.84)	23.00 (26.79)
Role-Emotional	72.28** (24.34)	74.55 (22.83)	72.60 (25.29)	73.95 (25.34)
Mental Health	74.04*** (18.70)	76.59 (17.03)	74.47 (17.69)	75.69 (17.61)

Notes: † Cells represent mean (standard deviation). ‡ The participating group refers to the sample that participated in the NRSP in the period from 2011 to 2018; the control group refers to the sample that didn’t participate in NRSP during the 2011–2018. § *, **, *** indicate the significance level of 10%, 5%, and 1%, respectively

Compared with those in 2011, all physical and mental health outcomes became worse as indicators of the elderly declined in 2018. The greatest reduction was in the bodily pain scores (-8.53 to -7.75). In 2011, compared with the older people in the control group, the older people in the insured group had significantly lower scores in physical functioning (-1.14, *p* < 0.01), bodily pain (-1.21, *p* < 0.05), general health (-2.49, *p* < 0.01), vitality (-1.55, *p* < 0.1), role-emotional (-2.27, *p* < 0.05), and mental health (2.55, *p* < 0.01). These findings indicated that the elderly with poorer HRQoL scores preferred to enrol in NRSP. In 2018, the difference between the insured group and the control group decreased. After enrolling in the NRSP, the physical functioning (0.52, *p* > 0.1) and role-physical (1.85, *p* < 0.01) scores of the insured group were higher than those of the control group in 2018.

### The effect of new rural social pension on the health-related quality of life of older people

**Fig. 2:**
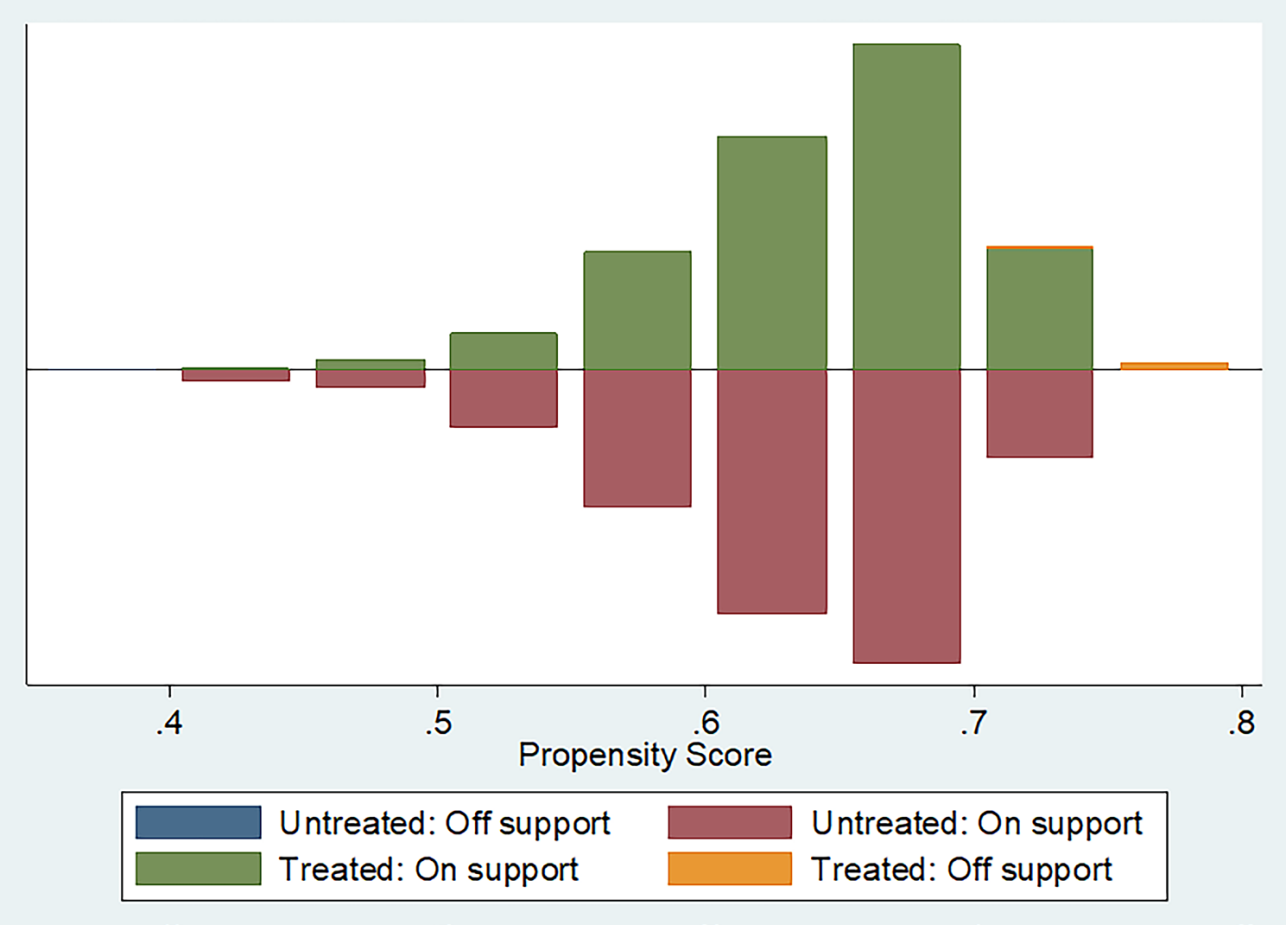
Density distribution of the propensity score after kernel matching for the insured and control groups

Figure 2 presents the density distribution of the propensity scores. The large overlap in propensity scores and similar distributions between the insured group and control group confirmed that the common support assumption was valid. The balance test for the PSM is shown in Table 3. Compared with the control group before matching, some characteristics of the insured group (including age, gender, marital status, educational attainment, smoking history, and region.) were significantly different. After the matching procedure, all the differences between the insured group and control group were nonsignificant, and the absolute standardized differences of covariate means (% bias) were less than 5%. Except for the number of children alive, there were substantial reductions in bias (ranging from 12.5 to 97.4%) indicating that the matched samples had relatively similar observational characteristics.

**Table 3:** The balance test of the propensity score matching (based on reference year 2011)

Variable	Matching	Insured	Control	*p*	%Bias	%Reduction Bias
Gender	Unmatched	0.47	0.43	0.05	7.2	
	Matched	0.46	0.45	0.32	3.1	56.9
Age	Unmatched	61.31	62.39	0.00	-15.2	
	Matched	61.38	61.43	0.84	-0.6	96.0
Marital status	Unmatched	0.87	0.82	0.00	14.2	
	Matched	0.87	0.88	0.53	-1.8	87.3
Educational attainment	Unmatched	0.37	0.40	0.05	-7.2	
	Matched	0.37	0.38	0.50	-2.1	71.3
Annual household income	Unmatched	7.53	7.34	0.22	5.6	
	Matched	7.53	7.51	0.85	0.7	86.8
Intergenerational support	Unmatched	2.11	2.11	0.92	-3.0	
	Matched	2.00	2.00	0.88	-0.1	97.4
Number of children alive	Unmatched	0.64	0.64	0.92	0.4	
	Matched	0.64	0.64	0.88	-0.5	-29.0
New rural cooperative medical system	Unmatched	0.95	0.95	0.95	-0.2	
	Matched	0.95	0.95	0.95	-0.2	12.0
Smoking history	Unmatched	0.41	0.36	0.01	9.7	
	Matched	0.41	0.38	0.14	4.6	52.3
Alcohol consumption history	Unmatched	0.31	0.31	0.92	0.4	
	Matched	0.31	0.31	0.92	0.3	12.5
Physical activity	Unmatched	0.76	0.77	0.50	-2.5	
	Matched	0.76	0.77	0.85	-0.6	76.1
Region	Unmatched	0.25	0.30	0.00	-11.2	
	Matched	0.25	0.26	0.62	-1.5	86.5

† *p* is the *p*-value from a t-test. ‡ % bias refer to absolute standardized difference of covariate means

Table 4 presents the effects of NRSP on HRQoL of the rural older people in China. Columns 1–4 report the effect of NRSP on the physical health status of the older people. According to the PSM-DID results (i.e., Treated$\times$Time), the NRSP significantly increased the physical functioning score (1.90, *p* < 0.01) and role-physical score (2.05, *p* < 0.05) of the rural older people. Although the NRSP decreased bodily pain (-1.80) and general health (-1.47), these decreases were insignificant at the 1% significance level.

**Table 4:** The effect of new rural social pension on health-related quality of life of the rural older people in China

	(1)	(2)	(3)	(4)	(5)	(6)	(7)	(8)
	Physical functioning	Role-Physical	Bodily Pain	General Health	Vitality	Social Functioning	Role-Emotional	Mental Health
Treated$\times$Time	1.90*** (0.71)	2.05** (0.87)	-1.80 (1.14)	-1.47 (1.54)	0.31 (1.85)	1.24 (2.15)	1.63 (2.31)	2.93** (1.47)
Treated	-2.67*** (0.47)	-1.53*** (0.49)	-0.85 (0.88)	-1.61 (1.19)	-1.98 (1.36)	-1.53 (1.56)	-2.10 (1.79)	-3.31*** (1.09)
Time	-2.36** (1.15)	-3.25*** (0.86)	-7.81*** (1.64)	2.45 (1.49)	-2.88 (1.80)	-1.20 (2.03)	-3.98* (2.21)	-2.06 (1.40)
Gender	1.67*** (0.42)	-0.19 (0.61)	6.03*** (0.72)	3.04*** (0.99)	6.67*** (1.09)	6.25*** (1.26)	-5.99*** (1.34)	4.62*** (0.93)
Age	-0.24*** (0.03)	-0.23*** (0.04)	-0.14 (0.75)	-0.13** (0.06)	-0.03 (0.06)	-0.11 (0.08)	-0.14* (0.75)	-0.07 (0.06)
Marital status	0.44 (0.44)	1.78*** (0.53)	-0.03 (0.74)	1.17 (0.90)	4.20*** (1.12)	1.02 (1.24)	-2.53** (1.24)	3.98*** (0.94)
Educational attainment	-0.84** (0.33)	-1.14*** (0.39)	-1.11* (0.58)	-0.48 (0.71)	-2.25*** (0.84)	-2.12** (0.97)	-5.20*** (0.98)	-1.43** (0.71)
Annual household income	0.17*** (0.06)	0.15** (0.08)	0.01 (0.09)	0.22 (0.14)	0.11*** (0.13)	0.15 (0.16)	0.06 (0.16)	0.01 (0.11)
Intergenerational support	-0.03 (0.04)	0.08 (0.05)	-0.18*** (0.07)	0.01 (0.09)	0.01 (0.10)	0.07 (0.12)	0.54*** (0.12)	0.07 (0.09)
Number of children alive	0.13 (0.34)	-0.34 (0.38)	0.44 (0.57)	-0.19 (0.72)	1.56* (0.84)	1.13 (0.97)	-0.13 (1.00)	0.89 (0.70)
NRCM	0.74 (0.95)	-0.65 (1.26)	-1.04 (1.29)	-2.19 (2.24)	-0.61 (2.32)	-4.58* (2.61)	0.44 (0.88)	1.27 (1.93)
Smoking history	-0.80* (0.45)	-0.08 (0.55)	-2.25*** (0.76)	-1.70 (1.05)	-1.59 (1.12)	-3.43** (1.35)	0.85 (1.46)	-1.71* (0.96)
Alcohol consumption history	0.91** (0.36)	1.04** (0.45)	-0.26 (0.63)	2.47*** (0.81)	2.12** (0.93)	1.41 (1.06)	0.24 (1.15)	0.63 (0.77)
Physical activity	-0.51 (0.33)	-0.51 (0.37)	1.21** (0.54)	0.46 (0.68)	1.89** (0.82)	2.02** (0.92)	-3.47*** (0.95)	1.82*** (0.66)
Region	0.84** (0.35)	1.02** (0.40)	5.46*** (0.55)	5.90*** (0.79)	6.04*** (0.86)	8.12*** (0.98)	1.99* (1.05)	6.17*** (0.74)
Constant	89.11*** (2.25)	110.30*** (2.91)	88.86*** (3.37)	63.17*** (4.48)	73.61*** (5.24)	78.05*** (5.88)	41.66*** (6.02)	72.39*** (4.27)

Notes: † Cells represent coefficient (robust standard error) of PSM-DID model. ‡ *, **, *** indicate the significance levels of 10%, 5%, and 1%, respectively

Columns 5–8 report the effect of NRSP on the mental health status of the older people. The NRSP significantly increased self-rated mental status (i.e., the score for feeling peaceful, happy, and calm) by 2.93 at a 0.5% significance level. There were no significant changes due to NRSP in the vitality, social functioning, and role-emotional status of the rural older people.

Most of covariates significantly impact the HRQoL of the rural elderly participants. The coefficient of gender, marital status, educational attainment, smoking history, alcohol consumption history, and region indicated that men who were married, lacked formal education, did not smoke, did not drink alcohol, and lived in the eastern area were more likely to have better physical and mental health status. The mean change in HRQoL per unit of age and annual household income indicated that relatively older and higher-income older people were more likely to have a better quality of life.

### The effect of new rural social pension on the health-related quality of life of older people in different subgroups

The impact of the NRSP on the HRQoL of the older people is shown in Table 5 by enrolment period, gender, age, level of education, marital status, annual household income, and area. After dropping the elderly who had enrolled in NRSP for less than two years or five years, the NRSP still exhibited significant effects on physical functioning (1.81, *p* < 0.05; 1.62, *p* < 0.05, respectively), role-physical (1.99, *p* < 0.05; 1.99, *p* < 0.05, respectively), and mental health (2.66, *p* < 0.1; 2.61, *p* < 0.1, respectively).

**Table 5:** The effect of new rural social pension on health-related quality of life of the rural older people with different pension enrolment times and socioeconomic characteristics

		Physical Functioning	Role-Physical	Bodily Pain	General Health	Vitality	Social Functioning	Role-Emotional	Mental Health
Enrollment time of the NRSP	$\ge$2 years	1.81** (0.75)	1.99** (0.88)	-1.82 (1.16)	-1.96 (1.56)	0.29 (1.88)	1.83 (2.33)	1.03 (2.18)	2.66* (1.50)
	$\ge$5 years	1.62** (0.76)	1.99** (0.93)	-1.81 (1.22)	-1.75 (1.64)	0.53 (1.97)	1.36 (2.45)	0.51 (2.31)	2.61* (1.58)
Gender	Male	2.14 (1.38)	4.66** (2.10)	-2.26 (2.09)	-0.65 (2.91)	-2.23 (3.33)	0.30 (4.36)	-5.56 (4.14)	3.70 (2.70)
	Female	1.87** (0.86)	1.20 (1.02)	-1.76 (1.43)	-1.54 (1.91)	1.89 (2.36)	2.45 (2.84)	4.11 (2.60)	3.29* (1.82)
Age	$$ <$$70	0.84 (0.79)	0.26 (0.93)	-1.26 (3.09)	-3.09 (1.97)	-0.78 (2.07)	1.60 (2.79)	-3.13 (2.54)	1.45 (1.78)
	$\ge$70	4.00** (1.61)	6.16*** (1.80)	-3.48** (1.39)	-1.51 (2.97)	3.03 (3.83)	2.02 (4.65)	11.59*** (4.21)	7.21** (3.04)
Educational attainment	Non-education	2.33** (1.11)	3.12** (1.43)	-2.54 (1.65)	-2.31 (2.21)	-1.52 (3.01)	-1.86 (3.61)	-2.57 (3.53)	2.89 (2.28)
	Have education	1.62* (0.94)	0.57 (1.19)	-0.49 (1.82)	0.73 (2.53)	1.46 (2.31)	4.40 (3.04)	3.95 (2.66)	2.78 (1.86)
Marital status	Married	1.32* (0.77)	1.64* (0.93)	-2.97** (1.28)	-1.30 (1.76)	-0.28 (2.07)	2.18 (2.60)	0.22 (2.44)	3.23** (1.62)
	Otherwise	3.52** (1.68)	3.62* (2.18)	1.77 (2.40)	-2.39 (3.41)	2.28 (4.24)	-2.20 (5.24)	4.71 (4.71)	2.98 (3.34)
Annual household income	$$ <$$50%	2.24** (1.12)	3.12** (1.43)	-2.54 (1.65)	-2.31 (2.21)	-0.54 (2.70)	5.07 (3.23)	0.49 (3.18)	-0.19 (2.09)
	$\ge$50%	1.12 (1.05)	0.57 (1.19)	-0.49 (1.82)	0.73 (2.53)	0.06 (2.94)	-2.11 (3.65)	2.70 (3.46)	6.31*** (2.27)
Region	Easter Area	0.67 (1.32)	0.87 (1.65)	-1.53 (1.79)	1.53 (3.19)	1.58 (3.10)	4.09 (4.06)	-2.34 (4.09)	0.55 (2.68)
	Middle and West Area	2.28*** (0.85)	2.52** (1.03)	-1.88 (1.45)	-2.70 (1.72)	-0.20 (2.32)	-0.44 (2.79)	2.64 (2.53)	3.83** (1.76)

Notes: † Cells represent coefficient (robust standard error) of PSM-DID model. ‡ The control variables include: gender, age, marital status, education level, annual household income (log), intergenerational support (log), number of children alive, medical insurance, smoking history, alcohol consumption history, physical activity, and region. § *, **, *** indicate the significance levels of 10%, 5%, and 1% respectively

The NRSP had a higher and more significant impact on role-physical status for men (4.66, *p* < 0.05) than for women (1.2, *p* > 0.1), while the NRSP had a greater impact on the physical functioning and mental health scores of female older people than on those of male older people. For various marital statuses and income categories, the NRSP had a greater and more significant impact on the physical functioning and role-physical status of the older people who were not married (3.52, *p* < 0.05; 3.61, *p* < 0.1, respectively) and had lower annual household incomes (2.24, *p* < 0.05; 3.12, *p* < 0.05, respectively). While this was going on, the NRSP had a bigger and more substantial impact on the mental health of the elderly who were married (3.23, *p* < 0.05) and had high annual household incomes (6.31, *p* < 0.01).

Compared with the person aged younger than 70 years, older people who are equal to or older than 70 years were disproportionately and considerably affected by the NRSP. The NRSP considerably enhanced the physical functioning (4.00, *p* < 0.05), role-physical (6.16, *p* < 0.01), role-emotional (11.59, *p* < 0.01), and mental health (7.25, *p* < 0.05) of older people equal to or above 70 years, although it significantly increased their bodily discomfort (-3.48, *p* < 0.05). The NRSP also had more pronounced and proportionately greater positive benefits on the physical functioning, role-physical, and mental health scores of the elderly without formal education who resided in the central and western regions.

### Robustness tests

We performed two sets of robustness tests (Additional file STable 2 and STable 3). In the first robustness check, we used the 0.1, 0.08, 0.04, and 0.02 bandwidths to estimate the effect of the NRSP on the HRQoL of the rural elderly. In the second robustness test, we changed the kernel matching method to k-nearest neighbours matching and radius matching methods. The results of these robustness tests were all similar to the main results in this study.

## Discussion

In this study, we discovered that the NRSP significantly and favourably impacted the physical functioning, role-physical health, and self-rated mental health of the rural older people. The long-term effects of the NRSP remained significant and favourable, although the health consequences of the program slightly declined. For those with the following characteristics: age equal to or over 70, no formal education, and living in the middle or western regions of China, the NRSP had more substantial and larger favourable health impacts on physical and mental health.

This study is the first to assess how social pension affects rural older residents’ HRQoL. The positive impacts of the NRSP on HRQoL of the older people indicate its support for health aging in China. The NRSP not only helped the older people be free of disease or infirmity, but also contributed to the process of developing and maintaining functional ability to enable wellbeing in older age [41]. The NRSP had positive benefits on rural elderly’s physical functioning, which was consistent with the findings of most extant research. For instance, Xu and Liu discovered that the NRSP could considerably increase older people’s independence in ADLs [3]. The NRSP has positive impacts on the older people’s physical health in part because it affects their labour participation status. According to related research, the NRSP might help the elderly in rural areas perform fewer labour-intensive tasks, such as farming, which would lower their risk of developing chronic illnesses and enhance their quality of life [42, 43]. Additionally, by raising the older people’s disposable income, the NRSP may improve their physical health. The additional income from the NRSP, similar to social pensions established in other nations, could result in better self-rated economic situations, improved nutritional intake and living conditions (such as toilet conditions and other health systems), more leisure activities, and informal care [16, 21, 44, 45].

In line with the findings of Pan et al., our study demonstrated that the NRSP significantly improved the mental health of the older people [22]. With the implementation of the NRSP, social pensions have replaced children as the main source of support of the older people. Fewer depression symptoms are caused by the older people having more financial independence and being more likely to live alone [16, 46]. The NRSP could also directly prevent the elderly from becoming depressed and anxious as a result of insufficient money to buy necessities [47]. In addition, the NRSP may improve emotional support and communication between generations, which can reduce older people depression rates [48].

Additionally, after nine years of universal coverage, this study is the first to quantify the long-term health effects of the NRSP. Prior research has employed only short-term panel datasets or data that have not yet included all of China’s provinces (i.e., prior to the 2012 universal implementation of the NRSP) [3, 13, 22]. While Xu and Liu examined the variations in the health effects of the NRSP for various enrolment dates, they contrasted only the entire sample with samples devoid of people who had only been involved in the program for six and twelve months [3].

The effect of the NRSP varying according to the socioeconomic characteristics of the older people is a further noteworthy observation. The results of the heterogeneity study demonstrated that disadvantaged groups (i.e., without formal education, low income, and living in the central and western regions), which often have relatively low health conditions, are more significantly affected by the NRSP. As a result, the NRSP reduced disparities in health among various populations and geographic areas. The possible reason for the differences in these subgroups is that they have relatively lower levels of per capita income and the marginal effect of the same amount of pension income on these disadvantaged groups was greater [13].

The Chinese government needs to consider the current study’s policy consequences. First, the Chinese government is able to continue promoting NRSP in rural areas because of the program’s long-term, significant positive effects on the health of older rural residents. Second, additional social supports need to be introduced in rural China because of the NRSP’s negligible effect on some components of HRQoL (such as physical discomfort and social functioning). Several measures, including free physical exams, long-term health care insurance, and community health services, could further enhance the elderly population’s overall quality of life [32, 49]. Third, the basic pensions of the NRSP may further increase given that the replacement rate for the non-contributory social pension of the NRSP (approximately 12% of average per capita net income) was lower than the average level of the Organization for Economic Co-operation and Development countries (approximately 30% of average wages) [50]. Fourth, the NRSP should be appropriately tilted towards the vulnerable groups (e.g., extra subsidies), in order to obtain greater health benefits.

The empirical findings concerning the effects of the NRSP may also offer useful lessons for decision-makers in other developing nations. Given that 56% of the rural population worldwide lacks access to health care, the NRSP and other social security support programs should be taken into consideration by policymakers in other developing nations [51]. For some low-income developing nations, the government-provided basic pension is essential for raising the enrolment rate. Although basic pensions cannot cover all living costs, the additional income may help the elderly in rural areas maintain or improve their health. Additionally, the diverse policy impacts of the NRSP motivate policymakers to consider equality when developing and administering social pensions, particularly for middle-income developing countries. Governments could offer larger basic pensions or allowances to enrolees with low incomes who are childless or reside in underdeveloped areas.

This study offers certain advantages. First, using the CHARLS dataset, we calculated the effects of the NRSP on older Chinese rural residents. Consequently, the results of this study representative because CHARLS is a high-quality public micro-database and nationally representative. Second, we performed a causal inference study utilizing the PSM-DID methodologies and robustness tests. With these techniques, self-selection bias was lessened, and individual and time-fixed effects were controlled. Finally, this study examined the long-term effects of policy and carried out a heterogeneity analysis, which added to the supporting data for social pension plans. Policymakers are able to focus more on disadvantaged groups because of the differential effects of policies on various subgroups.

Consideration should be given to a number of study limitations. The loss to follow-up and the missing values for some variables made it impossible to prevent the self-selection bias completely. This could result in an over- or underestimation of the effects of policies. The SF-36 utilized in this study has also been validated, but unobserved confounding remains because the CHARLS surveys do not ask the same questions as the original SF-36 and lack thorough depression-related data [32].

## Conclusion

The physical functioning, role-physical health, and self-rated mental health of the older people in rural areas were significantly improved by the NRSP in the long term. The NRSP had larger and more significant health effects on relatively vulnerable groups. The NRSP serves as a model for emerging nations seeking to raise the quality of life for rural older residents and reduce health disparities. In the future, a higher level of basic pension in the NRSP and additional supplemental programs that might enhance the overall HRQoL of older Chinese rural residents will be needed.

### Electronic supplementary material

Below is the link to the electronic supplementary material.

Supplementary Material 1

## Data Availability

The datasets used and analysed in this study are available in the CHARLS repository, https://charls.pku.edu.cn/en.

## Abbreviations

ADL: activities of daily living
ATT: average treatment effect on the treated
BPEE: Basic Pension for Enterprise Employees
CHARLS: China Health and Retirement Longitudinal Study
DID: difference-in-differences
HRQoL: health-related quality of life
NRSP: new rural social pension
PSM: propensity score matching
