# Supplementary material for: The effect of social pension on health-related quality of life of the rural older people: a panel study from China
Source: BMC Geriatr. 2024 Mar 27;24:291. doi: 10.1186/s12877-024-04880-y (PMC10976778; doi:10.1186/s12877-024-04880-y)
Supplement: Supplementary file 1 — Supplementary Material 1 [file 12877_2024_4880_MOESM1_ESM.docx]

**STable 1** The CHARLS variables used for measuring the health-related quality of life

| **SF-36 Scales** | **CHARLS Variables** | **Survey Questions** |
| --- | --- | --- |
| Physical functioning | DB001 | Do you have any difficulty with running or jogging about 1 Km? |
|  | DB002 | Do you have difficulty with walking 1 km? |
|  | DB003 | Do you have difficulty with walking 100 meters? |
|  | DB004 | Do you have difficulty with getting up from a chair after sitting for a long period? |
|  | DB005 | Do you have difficulty with climbing several flights of stairs without resting? |
|  | DB006 | Do you have difficulty with stooping, kneeling, or crouching? |
|  | DB007 | Do you have difficulty with reaching or extending your arms above shoulder level? |
|  | DB008 | Do you have difficulty with lifting or carrying weights over 10 jin, like a heavy bag of groceries? |
|  | DB009 | Do you have difficulty with picking up a small coin from a table? |
| Role-Physical | DB016 | Because of health and memory problems, do you have any difficulties with doing household  chores? |
|  | DB017 | Because of health and memory problems, do you have any difficulties with preparing  hot meals? |
|  | DB018 | Because of health and memory problems, do you have any difficulties with shopping  for groceries? |
|  | DB020 | Does anyone help you take medications? |
| Bodily Pain | DA041 | Are you often troubled with any body pains? |
|  | DA042 | On what part of your body do you feel pain? |
| General Health | DA001 | Would you say your health is excellent, very good, good, fair, or poor? |
|  | DA002 | Would you say your health is very good, good, fair, poor or very poor? |
| Vitality | DC015 | My sleep was restless |
|  | DC018 | I could not get ”going” |
| Social Functioning | DA056 | Have you done any of these activities in the last month? |
|  | DA057 | How often in the last month [did/have][you] do any of these activities? |
| Role-Emotional | DC010 | I had trouble keeping my mind on what I was doing |
|  | DC012 | I felt everything I did was an effort |
| Mental Health | DC009 | I was bothered by things that don’t usually bother me |
|  | DC011 | I felt depressed |
|  | DC013 | I felt hopeful about the future |
|  | DC014 | I felt fearful |
|  | DC016 | I was happy |
|  | DC017 | I felt lonely |

**STable 2** The effect of new rural social pension on health related quality of life of the rural elderly in China using different bandwidths

|  | 0.1  bandwidth | 0.08 bandwidth | 0.04 bandwidth | 0.02 bandwidth |
| --- | --- | --- | --- | --- |
| Physical functioning | 1.88*** (0.71) | 1.88*** (0.71) | 1.86*** (0.71) | 1.86*** (0.71) |
| Role-Physical | 2.05** (0.87) | 2.05** (0.87) | 2.03** (0.87) | 2.03** (0.87) |
| Bodily Pain | -1.79 (1.14) | -1.79 (1.14) | -1.82 (1.14) | -1.82 (1.14) |
| General Health | -1.47 (1.54) | -1.47 (1.54) | -1.53 (1.54) | -1.53 (1.54) |
| Vitality | 0.31 (1.85) | 0.31 (1.85) | 0.26 (1.85) | 0.26 (1.85) |
| Social Functioning | 1.63 (2.31) | 1.63 (2.31) | 1.53 (2.31) | 1.53 (2.31) |
| Role-Emotional | 1.25 (2.15) | 1.25 (2.15) | 1.16 (2.15) | 1.16 (2.15) |
| Mental Health | 2.93** (1.47) | 2.93** (1.47) | 2.93** (1.47) | 2.93** (1.47) |

Notes: † Cells represent coefficient (robust standard error) of the PSM-DID model. ‡ *, **, *** indicate the significance level of 10%, 5%, and 1%, respectively.

**STable 3** The effect of new rural social pension on health related quality of life of the rural elderly in China using different matching methods

|  | K-nearest Neighbors Matching | Radius Matching | |
| --- | --- | --- | --- |
| Physical functioning | 1.88*** (0.71) | 1.85*** (0.71) |  |
| Role-Physical | 2.05** (0.87) | 2.01** (0.87) |  |
| Bodily Pain | -2.16 (1.64) | -1.87 (1.14) |  |
| General Health | -1.47 (1.54) | -1.62 (1.54) |  |
| Vitality | 0.31 (1.85) | 0.24 (1.86) |  |
| Social Functioning | 1.63 (2.31) | 1.69 (2.31) |  |
| Role-Emotional | 1.25 (2.15) | 1.22 (2.16) |  |
| Mental Health | 2.93** (1.47) | 2.92** (1.48) |  |

Notes: † Cells represent coefficient (robust standard error) of the PSM-DID model. ‡ *, **, *** indicate the significance level of 10%, 5%, and 1%, respectively.
